# Cluster and network analysis of non-headache symptoms in migraine patients reveals distinct subgroups based on onset age and vestibular-cochlear symptom interconnection

**DOI:** 10.3389/fneur.2023.1184069

**Published:** 2023-05-26

**Authors:** Hui Li, Xiaonuo Xu, Jiying Zhou, Liang Dong

**Affiliations:** Department of Neurology, The First Affiliated Hospital of Chongqing Medical University, Chongqing, China

**Keywords:** migraine, non-headache symptoms, cluster analysis, network analysis, onset age of migraine, cochlear symptoms

## Abstract

**Objective:**

The present study endeavors to identify natural subgroups of migraine patients based on the patterns of non-headache symptoms, utilizing cluster analysis. Subsequently, network analysis was performed to estimate the structure of symptoms and explore the potential pathophysiology of these findings.

**Method:**

A total of 475 patients who met the diagnostic criteria for migraine were surveyed face-to-face during the period of 2019 to 2022. The survey included collecting demographic and symptom data. Four different solutions were generated by the K-means for mixed large data (KAMILA) clustering algorithm, from which the final cluster solutions were selected based on a series of cluster metrics. Subsequently, we performed network analysis using Bayesian Gaussian graphical models (BGGM) to estimate the symptom structure across subgroups and conducted global and pairwise comparisons between structures.

**Result:**

Cluster analysis identified two distinct patient groups, and the onset age of migraine proved to be an effective characteristic differentiating the two patient groups. Participants assigned to late-onset group showed a longer course of migraine, higher frequency of monthly headache attacks, and greater tendency toward medication overuse. In contrast, patients in early-onset group exhibited a higher frequency of nausea, vomiting, and phonophobia compared to their counterparts in the other group. The network analysis revealed a different symptom structure between the two groups globally, while the pairwise differences indicated an increasing connection between tinnitus and dizziness, and a decreasing connection between tinnitus and hearing loss in the early-onset group.

**Conclusion:**

Utilizing clustering and network analysis, we have identified two distinct non-headache symptom structures of migraine patients with early-onset age and late-onset age. Our findings suggest that the vestibular-cochlear symptoms may differ in the context of different onset ages of migraine patients, which may contribute to a better understanding of the pathology of vestibular-cochlear symptoms in migraine.

## 1. Introduction

Migraine is a common neurological disorder that affects 14–15% of the global population and it is considered a leading contributor to years lived with disability, accounting for 4.9% of the global burden of ill health (1). While migraine is commonly associated with headache pain, it is considered to be a multisensory processing disorder (2) that can manifest in various non-headache symptoms, including nausea, photophobia, and phonophobia. These non-headache symptoms can also have significant impact on an individual’s daily life, leading to a range of comorbidities in psychiatry, such as nausea, vomiting (3), osmophobia (4) and vestibular symptoms (5).

Non-headache symptoms of migraine display non-random patterns and are affected by various factors, including demographic variables and features specific to the migraine condition. Research has indicated that particular symptoms are more prevalent in specific subgroups; for example, nausea is more commonly reported by women with lower incomes, while photophobia is more frequent in men with obesity and allodynia (6). Additionally, non-headache symptoms of migraines display a dynamic characteristic. A study has suggested that during a median follow-up time of 9 years, the prevalence of cochlear symptoms increased from 15 to 49% with the progression of vestibular migraine (7). The patterns of non-headache symptoms further emphasize the heterogeneous nature of migraines based on existing evidence. Gaining an understanding of the patterns among these symptoms may provide valuable insights into the underlying mechanisms of the disorder.

In recent years, the application of machine learning techniques in headache research has garnered increasing attention. A classification-based approach has demonstrated promising outcomes in the differentiation of migraine subgroups through the clinical, imaging measures or the combines (8). The Random Forest classification approach, for example has demonstrated its remarkable ability to accurately diagnose primary headaches (9) and predict the effectiveness of medication in patients (10). Meanwhile, unsupervised methods, including cluster analysis, have also been employed in the study of headaches, such as cluster headache (11) and medication overuse headache (12), to facilitate a comprehensive understanding of the disease subgroups by identifying discriminatory factors. The machine learning techniques hold the potential to make valuable contributions to the diagnosis, treatment, and overall management of Migraine.

Moreover, network analysis has emerged as an increasingly popular method for investigating the interrelationships between symptoms or behaviors, with applications in various fields including psychiatry (13) and social science (14). This approach offers a novel perspective and a set of tools for comprehending the structure of the elements in the network, where the nodes represent elements, and the edges denote their associations. The width of the edges is indicative of the strength of the associations and provides a mechanism for exploring the structure and elucidating differences between structures. In another word, network analysis could prove valuable in exploring patterns of non-headache symptoms in migraine.

In the present study, we employ a cluster analysis of demographic and symptom data, to discern distinct patterns of non-head symptoms among migraine patients. Subsequently, we applied a network approach to gain deeper insights into the structure of the symptom network, hoping to elucidate its complex organization.

## 2. Method

### 2.1. Participants

This research was carried out from 2019 to 2022. The participants were selected from those who sought diagnosis, treatment, or services at the Neurological Clinic of the First Affiliation Hospital of Chongqing Medical University. A total of 485 participants who met the following inclusion criteria were recruited for the study: (1) Fulfilling the diagnostic criteria of Migraine based on the International Classification of Headache Disorders 3 (15); (2) Age ranging from 16 and 65 years old; (3) Absence of secondary headache disorders, severe neurological and psychological disorders. Participants were excluded based on the following exclusion criteria: (1) Course of migraine less than 1 year (*n* = 5); (2) frequency of migraine attacks less than once per month (*n* = 4); (3) the presence of errors in the collected data (*n* = 1). Eventually, the study was able to recruit a total of 475 participants.

### 2.2. Data collection

The present study utilized a survey to collect information regarding demographic data, migraine attack characteristics, and symptoms during migraine attacks. The survey was administered via face-to-face interviews with experienced clinicians and participants.

Our analysis focused on variables identified to be related to migraine non-headache symptoms in the literature, such as gender differences (16), the presence of family history and headache onset age (17) and the course (7). Our evaluation also considered the frequency and severity of migraine attacks and their association with non-headache symptoms, such as dizziness/vertigo (18). Additionally, we assessed medication overuse as a risk factor for migraine progression (19). The symptoms we included were those found previously associated with migraine (20–24). In selecting our final list of variables, we considered factors like sample size, missing data, and measurement quality. Our analysis included the following variables: Demographic data include gender, age, the onset age of migraine, the course of migraine, the family history of headache and dizziness, as well as an assessment of medication overuse, defined as the use of analgesic for more than 15 days per month. Migraine attack characteristics include the frequency and intensity of migraine attacks. In this study, symptoms were defined as those commonly experienced by the participants at least once during migraine attacks which include nausea, vomiting, photophobia, phonophobia, osmophobia, dizziness, vertigo, tinnitus, hearing loss, aural fullness, and visual symptoms such as blurred vision.

### 2.3. Data preprocessing

This study utilized the *mice* package (version 3.15.0) in R 4.2.2 to address the issue of missing data, which ranged from 0.2 to 1.9%. The missing values were imputed using the Predictive Mean Matching (PMM) method (25). Five imputed datasets were generated, and the average of the imputed data was calculated and rounded to replace the missing values. The variable representing age was not included in the following analysis due to its redundancy in providing additional information when onset age and course were considered.

### 2.4. Cluster analysis

#### 2.4.1. KAMILA clustering

The cluster analysis was performed using the KAMILA algorithm implemented in the R package KAMILA (version.1.2) (26), which is a model-based algorithm designed to cluster datasets containing both numerical and categorical variables. In a recent comparative study of nine clustering methods for heterogeneous data, the KAMILA algorithm was found to be dominant over the other methods, demonstrating stability and high performance across various simulated scenarios (27).

The KAMILA algorithm commences by initializing centroids for the continuous variables and parameters for the categorical variables. Subsequently, it calculates the Euclidean distance between the continuous variables and their nearest centroid, and an estimated mixture distribution of the continuous variables is derived from these distances. Regarding the categorical variables, the probabilities of observing the data, given the cluster, are computed, and the log-likelihood of the sum of these two components is utilized to allocate each subject to a cluster. The centroids and parameters are updated to better depict the clusters, and this process is reiterated until cluster stability is achieved. Details are described elsewhere (28).

Prior to conducting the analysis, the continuous variables were standardized by cantering them to a mean of 0 and a standard deviation of 1. Data was fit to the model with 25 random initializations and a maximum of 25 iterations per initialization, which is considered sufficient to produce stable results (28). The number of clusters was selected based on the prediction strength method (29). The threshold was chosen to 0.8. the number of cross-validation runs was set to 10 and the average predict strength was calculated.

The stability of the clusters was evaluated using the Jaccard coefficient (30). This coefficient measures the similarity between two subsets of a set based on their set membership. To determine the stability of the clusters, new datasets were generated from the original dataset using bootstrapping and the KAMILA clustering method was applied to each of these datasets. For each cluster identified in the original clustering, the most similar cluster in the new clustering was identified, and the Jaccard coefficient was recorded. The stability of each individual cluster was assessed by taking the mean of the Jaccard coefficient over all resampled datasets. In this study, the number of bootstrapping runs was set to 2000 to obtain cluster stability result. The Jaccard index should be over 0.5 and preferred over 0.75 (30).

#### 2.4.2. Partial least squares-discriminant analysis

To gain a deeper understanding of the cluster result, a Partial Least Squares Discriminant Analysis (PLS-DA) implemented in the R package mdatools (version 0.13.1) (31) was conducted. Partial Least Squares Discriminant Analysis (PLS-DA) is a multivariate analysis technique used to investigate the relationship between predictor variables and a categorical response variable. The remaining variables were set as predictors, with the cluster class designated as the responder. The model was constructed using a maximum of five components and cross-validation was performed 10 times to obtain the classification accuracy. Classification accuracy measures the percentage of correctly classified instances in the dataset. A high accuracy score suggests that the RLS-DA model has properly divided the dataset in general.

The first two components of the PLS-DA model were used to visualize the clustering analysis results in two dimensions, as they captured most of the variation. The clusters were separated, and patterns within the data were identified by plotting the observations on these two components.

Furthermore, the VIP (Variable Importance in Projection) score was calculated for each variable included in the PLS-DA model. The VIP score provides a measure of the relative importance of each variable in predicting the response variable. It is calculated as the weighted sum of the squares of the PLS weights, where each weight represents the significance of the corresponding variable in the PLS model. VIP scores greater than 1 indicate strong importance of a variable in predicting the response variable.

The appropriate number of clusters was determined based on the evaluation of both cluster metrics and PLS-DA’s plot. A descriptive analysis was carried out using Wilcox-rank-sum test for numerical variables (assuming not follow a normal distribution) and chi-square tests for categorical variables. To address the issue of inflated Type I error rates due to multiple comparisons, the Bonferroni correction was employed to yield a new alpha level of 0.0026.

### 2.5. Network analysis

The next part of this study was to investigate the underlying structure of symptoms in different clusters, using the *BGGM* (Bayesian Gaussian Graphical Models) package (version 2.0.4) in R (32). The BGGM provided an innovative methodology for estimating network structures using a Bayesian approach. Details regarding the Bayesian approach are described elsewhere (33). The symptoms served as node and their non-zero partial correlations were denoted as edges.

The BGGM package provides two methods for comparing networks. The “global” method assesses the degree of difference in network structure by comparing the partial correlation matrix distance (CMD) of two networks and allows for an overall evaluation of the differences between the networks in terms of their structure (34). To evaluate the differences, the posterior predictive distribution is computed under the assumption of group equality, providing the expected error under the null model of equivalence between partial correlation matrices. The CMD is then estimated for the observed groups and compared to the posterior predictive distribution, from which a posterior predictive value of p is computed. Rejection of the null model indicates that the assumption of group equality is not valid. After establishing the global difference, the pairwise comparison method is used to evaluate the differences between the networks at the individual edge level, by comparing the posterior distributions of each edge in a similar way.

## 3. Result

### 3.1. Cluster analysis

**Figure 1:**
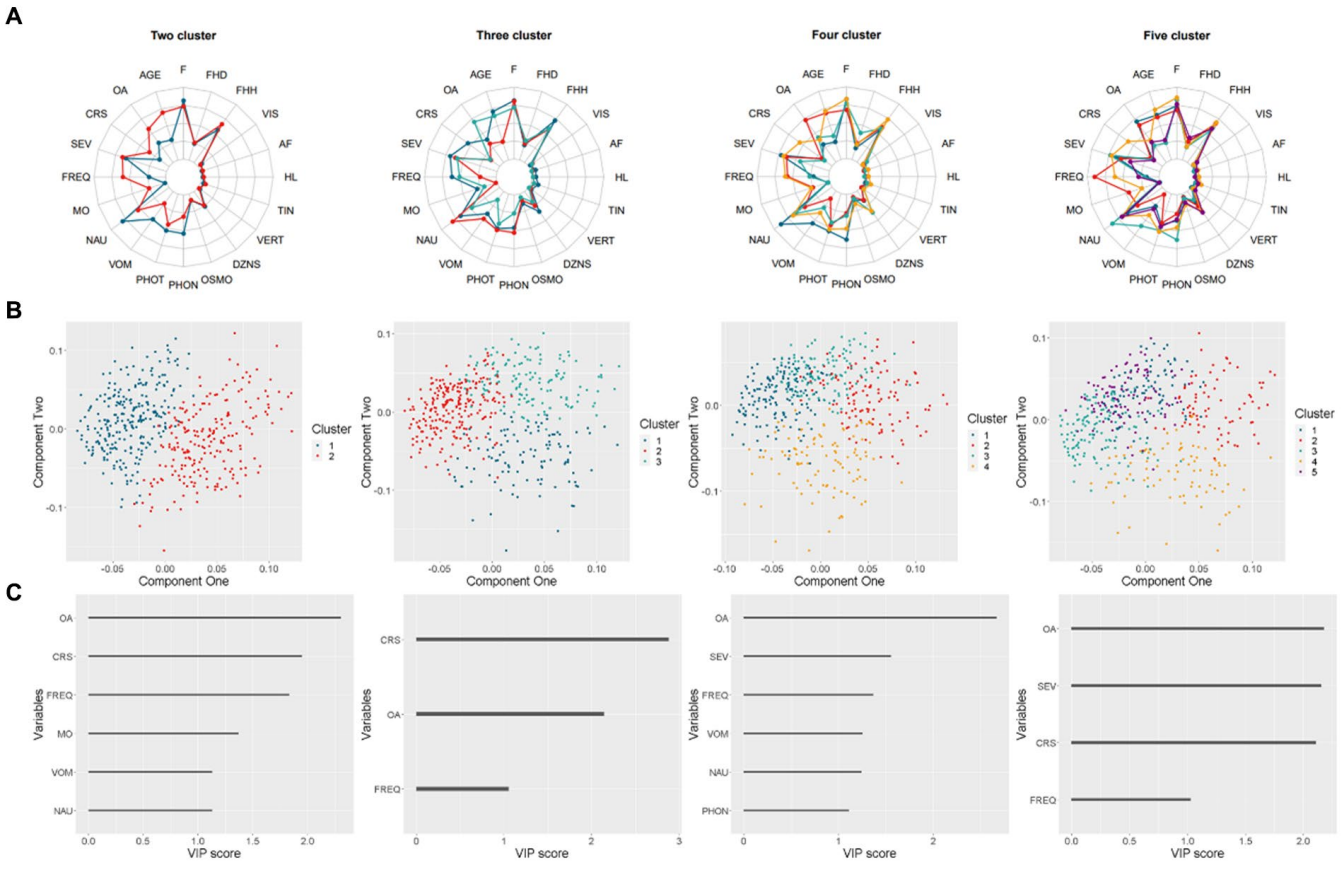
Results of the clustering analysis for two to five cluster solutions. **(A)** Radar charts representing the means on scaled values (binary variables were coded numerically). **(B)** Scatter plots displaying the distribution of four solutions. **(C)** Bar charts demonstrating the Variable Importance in Projection (VIP) score that exceeds one for each solution.

The KAMILA algorithm was employed to form two, three, four, and five clusters. The cluster predictive strength and Jaccard coefficients were calculated for each solution, as summarized in Supplementary Table S1. The two-cluster solution exhibited the highest predictive strength (0.860) compared to other solutions. Both clusters in the two-cluster solution showed high stability with Jaccard coefficients of 0.931 and 0.920, respectively. The cross-cluster difference was illustrated using radar plots in Figure 1A. The two-cluster and three-cluster solutions yielded the most distinct radar shapes when compared to other solutions.

### 3.2. Partial least squares-discriminant analysis

For each solution, a Partial Least Squares-Discriminant Analysis (PLS-DA) was performed, with the accuracy of two-cluster and three-cluster exceeding 0.9. The first two components were utilized to generate cluster patterns, as depicted in Figure 1B. The two-cluster solution exhibited superior separation compared to other solutions, which demonstrated varying degrees of overlap.

To identify the most important feature of each solution, the Variable Importance in Projection (VIP) score was calculated for each predictor. Predictors with a VIP score over 1 were plotted in Figure 1C. Notably, the onset age of migraine was ranked among the top 2 VIP predictors across all solutions. In the two-cluster and three-cluster solutions, which outperformed other solutions, the course of migraine was also ranked as top2 VIP predictors.

The findings above suggest that the two-cluster solution provides the most optimal grouping of participants. Demographic and symptoms characteristics of participants by cluster are presented in Table 1. There were differences in age, onset age, course, frequency of headache, medication overuse and the occurrence of nausea, vomit and phonophobia. Participants assigned to cluster two exhibit a later onset of migraine symptoms compared to those in cluster one, accompanied by a longer course of migraine, a higher frequency of headache attacks per month, and a greater potion for medication overuse. With regards to symptoms, participants belonging to cluster one demonstrates a greater frequency of nausea, vomiting, and phonophobia when compared to those in the other.

**Table 1:** Demographic and clinical characteristics of patients by cluster.

Characteristics	Clusters		*χ* ^2^	value of *p*
	One *N* = 260	Two *N* = 215
Female (%)	212 (44.6%)	158 (33.3%)	4.429	0.035
Age, median (IQR)	32 (25, 36)	53 (46.5, 56)		< 0.001*
Onset age, median (IQR)	22 (16, 28)	35 (27.5, 43)		< 0.001*
Course, median (IQR)	8 (4, 11)	18 (10, 29.5)		< 0.001*
Severity, median (IQR)	7 (5, 7)	7 (5, 8)		0.011
Frequency of headache, median (IQR)	5 (3, 10)	20 (6, 30)		< 0.001*
Chronic Migraine (%)	35	115	56.52	< 0.001*
Migraine with aura (%)	16	4	0.15	0.699
Medication Overuse (%)	6 (1.3%)	55 (11.6%)	56.952	< 0.001*
Family history of headache (%)	146 (30.7%)	142 (29.9%)	4.825	0.028
Family history of dizziness (%)	62 (13.1%)	54 (11.4%)	0.103	0.748
Symptoms				
Nausea (%)	208 (43.8%)	115 (24.2%)	38.013	< 0.001*
Vomit (%)	124 (26.1%)	44 (9.3%)	38.165	< 0.001*
Photophobia (%)	139 (29.3%)	96 (20.2%)	3.6544	0.056
Phonophobia (%)	140 (29.5%)	65 (13.7%)	26.749	< 0.001*
Osmophobia (%)	24 (5.1%)	19 (4%)	0.022	0.882
Dizziness (%)	67 (14.1%)	51 (10.7%)	0.264	0.607
Vertigo (%)	9 (1.9%)	4 (0.8%)	1.133	0.287
Tinnitus (%)	11 (2.3%)	16 (3.4%)	2.263	0.132
Hearing loss (%)	5 (1.1%)	7 (1.5%)	0.848	0.357
Aural fullness (%)	5 (1.1%)	7 (1.5%)	0.848	0.357
Vision symptoms (%)	17 (3.6%)	9 (1.9%)	1.259	0.262

* Indicating significant difference under the adjusted value of *p* 0.0022.

### 3.3. Network analysis

**Figure 2:**
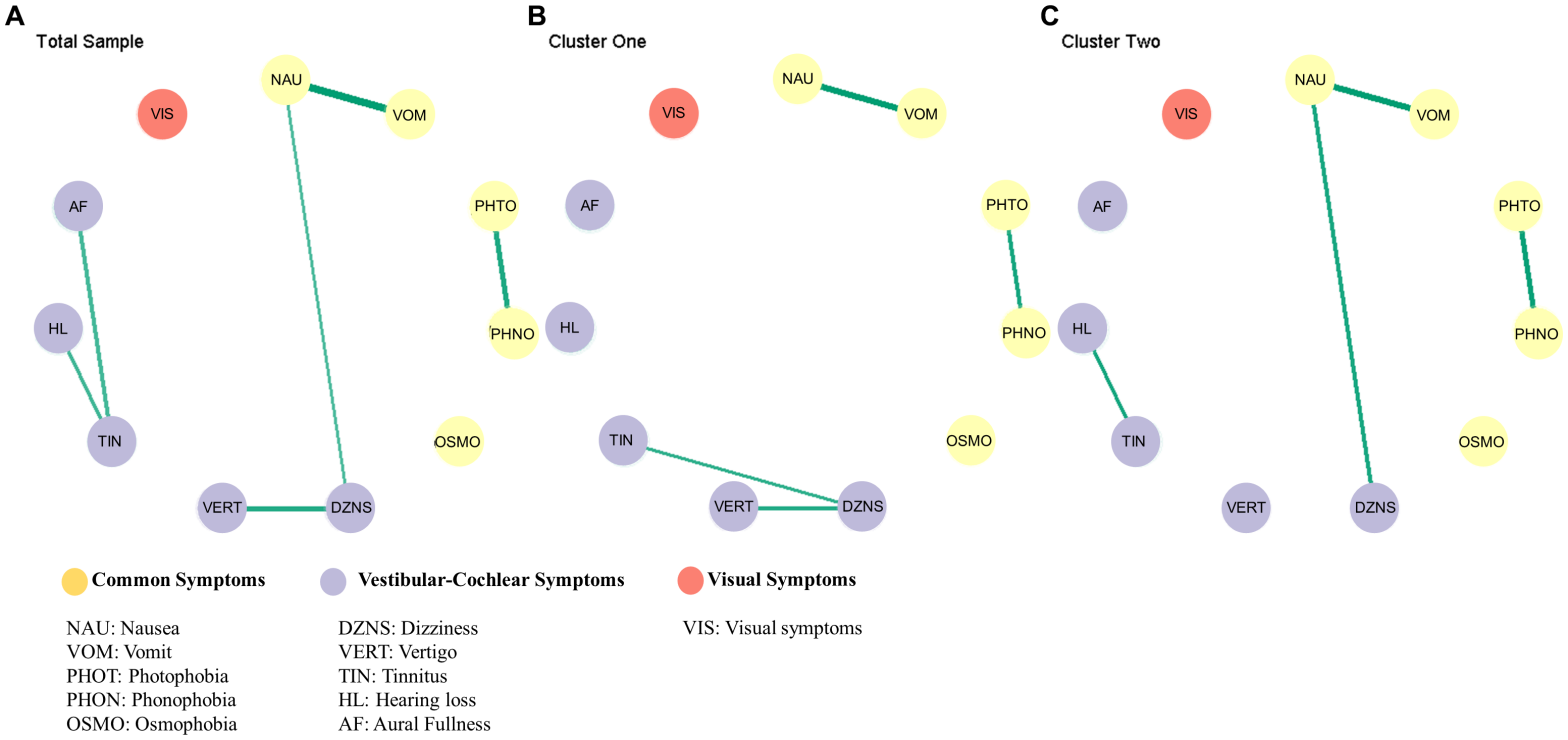
Results of the network analysis. Estimated symptoms network for total sample **(A)**, cluster one **(B)**, cluster two **(C)**.

The symptoms structure of all sample, cluster one, cluster two was estimate using Bayesian Gaussian graphical models in Figure 2.

The difference in the global network was evaluated by comparing the partial correlation matrix distance (CMD) of the two networks. The CMD between cluster one and cluster two symptom networks yielded a value of 0.57, with a significance level of *p* < 0.01, thus rejecting the null hypothesis of group equality. The results of the global test indicate that there was a significant difference in the network structure.

**Figure 3:**
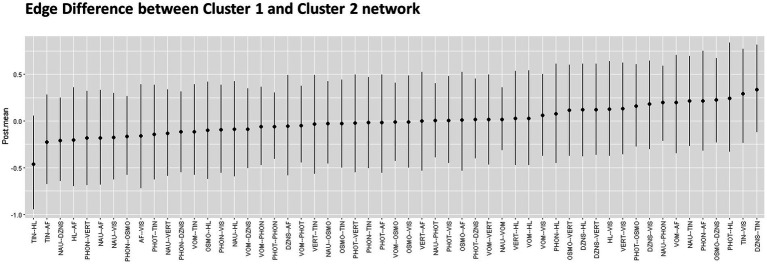
Pairwise edge differences in two networks represented with Posterior mean and 95% credential interval.

Figure 3 displays the posterior means and 95% credibility intervals for all possible edges. Within the 95% credibility interval, there were no significant differences in individual edges between the two clusters. However, if the conditions were relaxed, there were two potential candidates for significant differences. Specifically, within the 90% credibility interval, the connection between tinnitus and hearing loss exhibited a negative association in Cluster one compared to Cluster two. Within the 85% credibility interval, the connection between tinnitus and dizziness showed a positive association in Cluster one compared to Cluster two. All pairwise edge difference test results were documented in Supplementary Table S2.

## 4. Discussion

In this exploratory study, we employed machine learning techniques and network analysis to examine the relationships among non-headache symptoms in individuals with migraines. We discovered two distinct subgroups, with the early-onset group showing a positive connection between dizziness and tinnitus and a negative connection between tinnitus and hearing loss in comparison to the other group.

### 4.1. Early onset age of migraine

Numerous studies have identified a link between genetic predisposition and the early onset of migraine. Firstly, the family history of migraine has been associated with an early age of onset. For instance, a Finnish study of 4,930 individuals found that individuals who experienced migraine headaches before the age of 20 had a significantly higher mean polygenic risk score and stronger family history of migraine (35). Similarly, a large-scale study of 2,829 migraine patients corroborated this finding by demonstrating that a lower age of migraine onset was associated with a stronger family history of the condition, as confirmed by a validated web-based questionnaire (36).

Secondly, various studies have linked specific genes with the early onset of migraine. For example, one study examining the relationship between the common methionine/valine polymorphism at codon 129 within the prion protein gene (PRNP) and migraine revealed that patients with the PRNP 129VV genotype were significantly more likely to experience migraine at an earlier age (37). Another study focused on hemiplegic migraine and found that patients with mutations in CACNA1A, ATP1A2, or SCN1A tended to have a lower age at disease onset (38).

However, there are contentious findings regarding the relationship between early onset age and migraine. One study suggests that a stronger family history of migraine is associated with a lower age-at-onset, higher frequency and number of medication days, and the migraine with aura subtype (36). However, another study shows that family history was linked to an earlier age at onset, particularly in patients without aura (39). Moreover, in another study, the correlation between the early-onset group and a higher frequency of migraine attacks was not confirmed when adjusting for the years of illness (40).

Our hypothesis is that the inconsistency among these studies may be due to data collection at the time of migraine diagnosis, without considering the effect of the migraine course on the disease, as migraine might progress over time. In this current study, we used an unsupervised method to divide migraine patients based on both headache features and non-headache symptoms. We found that the onset age of migraine ranks first in dividing the group, followed by the course of migraine. We have successfully identified a group of early onset migraine patients with a relatively shorter course of illness. However, in the relatively weaker solution of three clusters, the sample can be further divided into early onset migraine patients with a longer course and late onset migraine patients with a shorter course. Therefore, it may be worthwhile to consider both onset age and the course of illness in future studies.

### 4.2. Tinnitus and dizziness in early onset migraine

Several studies have established a connection between early-onset migraine and tinnitus. A cross-sectional study of 5,729 participants found a positive correlation between migraine and tinnitus in young adults (22). Another survey, utilizing the 1999 to 2004 NHANES data, showed that among patients with tinnitus, those with migraine tended to be younger than those without migraine (21). Moreover, a genome-wide association study (GWAS) of the Han Chinese population revealed that rs146094041 in ESRRG and rs7124169 in chromosome 11 were more susceptible to early-onset migraine (41). The ESRRG expression is highly upregulated in cochlear hair cells and linked to a candidate gene for senile hearing impairment (42). Interestingly, a study investigating carotid intima-media thickness (cIMT) in children and adolescents of migraineurs compared to healthy controls found significantly thicker cIMT, despite similar biochemical profiles and glucose homeostasis, indicating a possible primitive vascular function abnormality in pediatric migraineurs (43). Another study, examining 820 males and 528 females, revealed significant associations between tinnitus and increased intima-media thickness after adjusting for confounding factors such as sex and age (44).

Regarding the early onset of migraines and vestibular symptoms, we conducted a search for evidence of vestibular migraines among children and adolescents. A recent study examined age-related characteristics in the onset of vestibular migraines and discovered that external spontaneous vertigo and visually-induced vertigo are distinguishing clinical features of juvenile forms (45). Another study investigating vestibular migraine in children revealed high rates of abnormalities in vestibular test, suggesting the involvement of the lateral semicircular canal, the utricle, and the superior vestibular conduction pathway in vestibular migraine of childhood (46), indicating peripheral vestibular dysfunction may contribute to the manifestation of vestibular symptoms.

A recent study has analyzed the auditory symptoms of patients with vestibular migraine and their connection to vestibular symptoms. The patients were divided into two groups based on the presence or absence of hearing loss. The study found that tinnitus is more common in patients with vestibular migraine and hearing loss. Additionally, the group with vestibular migraine and hearing loss had a younger age of onset compared to the other group (47).

In patients with migraine, it is theorized that the trigeminal nerve axons are abnormally activated, leading to the release of neurotransmitters including calcitonin gene-related peptide (CGRP) and substance P (SP), neurokinin A, and nitrous oxide. These then cause vasodilation, mast cell degranulation, and neurogenic inflammation in the inner ear, resulting in the subsequent impact on inner ear function (48, 49), which ultimately leads to the development of vestibular-cochlear symptoms. In animal studies, it has been observed that CGRP is associated with the maturity (50) and function (51) of the inner ear in mouse models. In human studies, it was found that the plasma level of CGRP is higher in young patients with migraines compared to those with other types of headaches and controls (52).

Based on the previous research and the results of this study, we believe that there is a certain degree of association between tinnitus and dizziness in patients with early-onset migraine. The exact reason for this association is not yet clear and may be related to genetics, blood vessels, inner ear function, and neural inflammatory factors. However, the evidence regarding the treatment of tinnitus in patients with migraines is still insufficient. One study suggest that migraine prophylaxis therapy with Flunarizine may be effective (53). Since anti-CGRP treatment appears to be effective in vestibular migraine (54), we recommend that its efficacy be further explored for early onset migraine patients with tinnitus.

### 4.3. Tinnitus and hearing loss in late onset of migraine

In the current investigation, while we were successful in identifying a subset of individuals with early onset migraine and a comparatively shorter disease duration, we encountered challenges in interpreting the findings of the other group. This group was also characterized by a higher frequency of migraine attacks and a greater proportion of medication overuse. Our limited sample size prevented us from conducting further subgroup analysis. The observed positive correlation between tinnitus and hearing loss in this group may be attributed to multiple factors.

Migraine patients have a higher incidence of tinnitus and subjective hearing loss (21), with sensorineural hearing loss occurring between the ages of 40 to 50 years (55). In a meta-analysis comprising six studies, the pooled hazard ratio of migraine with the risk of sensorineural hearing loss was slightly higher in people over 40 years of age (56).

The onset of migraine later in life has been associated with an elevated risk of stroke (57), while another study found that hearing loss patients are also more likely to experience stroke (58). While the link between late-onset migraine, hearing loss, and stroke remains unconfirmed, it is commonly believed to be related to vascular issues. Additionally, the growing connection between tinnitus and hearing loss may also be related to an increased risk of vascular problems, as both conditions can be influenced by vascular factors (59). Given the possible connection between these conditions, it is important for healthcare professionals to remain vigilant for signs of vascular conditions in patients with late-onset migraines and auditory symptoms. Further research is necessary to establish a definitive link, but in the meantime, increased awareness may lead to earlier detection and treatment.

### 4.4. Limitations

As far as we know, this study represents the first instance in which machine learning and network analysis have been utilized to investigate the interconnections among non-headache symptoms of migraine. However, this study is subject to certain limitations. Firstly, it is important to note that the data for this study were obtained through a convenient sampling method of consecutive patients. As such, we acknowledge that the generalizability of our findings may be limited to the population from whom we sampled. We suggest that further research should adopt more rigorous sampling techniques to obtain representative data and test whether our outcomes are transferrable to populations and settings beyond those of this study. Secondly, despite our effort to comprehensively assess non-headache symptoms experienced during migraine attacks and all related factors, we acknowledge that that we did not include all non-headache symptoms commonly seen in migraine patients, such as allodynia and kinesiophobia, nor did we evaluate other related factors. The lack of a unified scale to evaluate all the symptoms introduced certain limitations to our study. The use of specific scales such as the Allodynia Symptom Checklist (ASC) to evaluate allodynia would have been beneficial to improve the accuracy of our study. However, other symptoms such as phonophobia and photophobia are still assessed through consultation rather than standardized scales. Therefore, the non-exhaustive measurement of non-headache symptoms should be considered as a limitation of our study. Future research could benefit from the use of standardized scales to assess a broader range of non-headache symptoms in migraine patients. Thirdly, although we identified an overall structural difference across the two groups, the pairwise differences were only confirmed under relaxed conditions, likely due to the relatively small sample size. Lastly, we only analyzed non-headache symptoms during the ictal phase, which may not fully demonstrate the overall patterns of non-headache symptoms across the entire migraine attack. Further studies, with larger samples and the inclusion of interictal symptoms, are necessary to gain a more detailed understanding of symptom patterns.

## Conclusion

We have found two distinct non-headache symptoms structure of group with early onset age of migraine and the other utilizing cluster and network analysis. Our findings suggest that the vestibular-cochlear symptoms may differ in the context of different onset ages of migraine patients, hoping to gain a better understanding of the pathology of vestibular-cochlear symptoms in migraine.

## Data availability statement

The raw data supporting the conclusions of this article can be shared with other researchers on request.

## Ethics statement

Ethical review and approval was not required for the study on human participants in accordance with the local legislation and institutional requirements. Written informed consent to participate in this study was provided by the participants’ legal guardian/next of kin.

## Author contributions

HL and LD contributed to conception and design of the study. XX and HL collected the data and organized the database. HL performed the analysis and wrote the first draft of the manuscripts. All authors contributed to manuscript revision, read, and approved the submitted version.

## Conflict of interest

The authors declare that the research was conducted in the absence of any commercial or financial relationships that could be construed as a potential conflict of interest.

## Publisher’s note

All claims expressed in this article are solely those of the authors and do not necessarily represent those of their affiliated organizations, or those of the publisher, the editors and the reviewers. Any product that may be evaluated in this article, or claim that may be made by its manufacturer, is not guaranteed or endorsed by the publisher.
