# Supplementary material for: Cluster and network analysis of non-headache symptoms in migraine patients reveals distinct subgroups based on onset age and vestibular-cochlear symptom interconnection
Source: Front Neurol. 2023 May 26;14:1184069. doi: 10.3389/fneur.2023.1184069 (PMC10251495; doi:10.3389/fneur.2023.1184069)
Supplement: Supplementary file 1 [file Table_1.DOCX]

Supplementary Material

Network analysis of non-headache symptoms in migraine patients reveals distinct subgroups based on onset age and cochlear symptom interconnection

**Hui Li 1†, Xiaonuo Xu2†,Liang Dong1*, Jiying Zhou2***

*** Correspondence:**

Liang Dong

The First Affiliated Hospital of Chongqing Medical University, Department of Neurology, 400016, Chongqing, China

Dongliang_cqmu@163.com

Jiying Zhou

The First Affiliated Hospital of Chongqing Medical University, Department of Neurology, 400016, Chongqing, China

zheadache@163.com

Supplementary Data

Supplementary Material should be uploaded separately on submission. Please include any supplementary data, figures and/or tables.

Supplementary material is not typeset so please ensure that all information is clearly presented, the appropriate caption is included in the file and not in the manuscript, and that the style conforms to the rest of the article.

# Supplementary Tables

Table 1

|  | Cluster metrics | | PLS-DA metrics |
| --- | --- | --- | --- |
|  | Average Predictive strength | Jaccard coefficient | Accuracy |
| Two cluster | 0.860 | Cluster 1:0.931  Cluster 2:0.920 | Cluster 1:0.985  Cluster 2:0.985 |
| Three cluster | 0.759 | Cluster 1:0.703  Cluster 2:0.869  Cluster 3:0.700 | Cluster 1:0.945  Cluster 2:0.941  Cluster 3:0.918 |
| Four cluster | 0.479 | Cluster 1:0.541  Cluster 2:0.580  Cluster 3:0.312  Cluster 4:0.679 | Cluster 1:0.935  Cluster 2:0.931  Cluster 3:0.869  Cluster 4:0.943 |
| Five cluster | 0.431 | Cluster 1:0.531  Cluster 2:0.510  Cluster 3:0.471  Cluster 4:0.556  Cluster 5:0.298 | Cluster 1:0.819  Cluster 2:0.914  Cluster 3:0.855  Cluster 4:0.903  Cluster 5:0.787 |

Table 2

| Relation | Post.mean | Post.sd | Cred.lb | Cred.ub |
| --- | --- | --- | --- | --- |
| NAU--VOM | 0.016 | 0.172 | -0.313 | 0.357 |
| NAU--PHOT | 0.004 | 0.203 | -0.391 | 0.404 |
| VOM--PHOT | -0.048 | 0.210 | -0.448 | 0.375 |
| NAU--PHON | 0.198 | 0.206 | -0.215 | 0.588 |
| VOM--PHON | -0.060 | 0.210 | -0.473 | 0.362 |
| PHOT--PHON | -0.058 | 0.180 | -0.405 | 0.305 |
| NAU--OSMO | -0.026 | 0.225 | -0.458 | 0.425 |
| VOM--OSMO | -0.011 | 0.216 | -0.428 | 0.410 |
| PHOT--OSMO | 0.161 | 0.227 | -0.274 | 0.604 |
| PHON--OSMO | -0.163 | 0.213 | -0.575 | 0.262 |
| NAU--DZNS | -0.209 | 0.225 | -0.642 | 0.249 |
| VOM--DZNS | -0.088 | 0.216 | -0.508 | 0.348 |
| PHOT--DZNS | 0.014 | 0.219 | -0.403 | 0.450 |
| PHON--DZNS | -0.118 | 0.222 | -0.551 | 0.316 |
| OSMO--DZNS | 0.226 | 0.227 | -0.233 | 0.670 |
| NAU--VERT | -0.133 | 0.237 | -0.589 | 0.337 |
| VOM--VERT | 0.015 | 0.249 | -0.465 | 0.494 |
| PHOT--VERT | -0.024 | 0.273 | -0.548 | 0.495 |
| PHON--VERT | -0.180 | 0.255 | -0.685 | 0.319 |
| OSMO--VERT | 0.115 | 0.261 | -0.373 | 0.602 |
| DZNS--VERT | 0.121 | 0.247 | -0.364 | 0.610 |
| NAU--TIN | 0.212 | 0.249 | -0.271 | 0.695 |
| VOM--TIN | -0.117 | 0.245 | -0.579 | 0.390 |
| PHOT--TIN | -0.143 | 0.260 | -0.628 | 0.387 |
| PHON--TIN | -0.019 | 0.245 | -0.507 | 0.470 |
| OSMO--TIN | -0.026 | 0.245 | -0.500 | 0.439 |
| DZNS--TIN | 0.338 | 0.239 | -0.121 | 0.814 |
| VERT--TIN | -0.034 | 0.267 | -0.565 | 0.491 |
| NAU--HL | -0.089 | 0.260 | -0.595 | 0.423 |
| VOM--HL | 0.028 | 0.262 | -0.475 | 0.537 |
| PHOT--HL | 0.242 | 0.300 | -0.329 | 0.838 |
| PHON--HL | 0.079 | 0.273 | -0.449 | 0.610 |
| OSMO--HL | -0.101 | 0.270 | -0.622 | 0.421 |
| DZNS--HL | 0.119 | 0.254 | -0.380 | 0.611 |
| VERT--HL | 0.027 | 0.255 | -0.474 | 0.533 |
| TIN--HL | -0.461 | 0.256 | -0.948 | 0.054 |
| NAU--AF | -0.180 | 0.261 | -0.682 | 0.332 |
| VOM--AF | 0.199 | 0.268 | -0.348 | 0.702 |
| PHOT--AF | -0.015 | 0.271 | -0.555 | 0.493 |
| PHON--AF | 0.217 | 0.271 | -0.321 | 0.749 |
| OSMO--AF | 0.011 | 0.272 | -0.536 | 0.523 |
| DZNS--AF | -0.053 | 0.277 | -0.585 | 0.492 |
| VERT--AF | 0.002 | 0.277 | -0.534 | 0.522 |
| TIN--AF | -0.225 | 0.248 | -0.674 | 0.281 |
| HL--AF | -0.202 | 0.262 | -0.701 | 0.358 |
| NAU--VIS | -0.178 | 0.234 | -0.628 | 0.295 |
| VOM--VIS | 0.062 | 0.229 | -0.376 | 0.502 |
| PHOT--VIS | 0.005 | 0.241 | -0.452 | 0.478 |
| PHON--VIS | -0.095 | 0.240 | -0.554 | 0.387 |
| OSMO--VIS | -0.011 | 0.249 | -0.501 | 0.483 |
| DZNS--VIS | 0.182 | 0.243 | -0.303 | 0.645 |
| VERT--VIS | 0.132 | 0.249 | -0.359 | 0.623 |
| TIN--VIS | 0.290 | 0.259 | -0.236 | 0.773 |
| HL--VIS | 0.124 | 0.259 | -0.374 | 0.641 |
| AF--VIS | -0.162 | 0.280 | -0.718 | 0.392 |

**
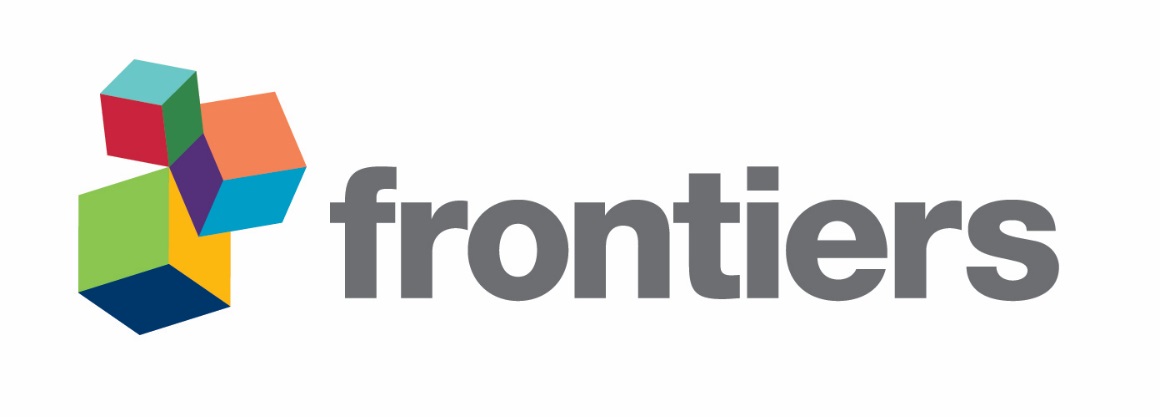
**
